# Successful carbon dioxide laser ablation in acquired idiopathic vulval lymphangiectasia

**DOI:** 10.1093/skinhd/vzag094

**Published:** 2026-06-25

**Authors:** Brent James Doolan, Emma Craythorne, Fiona Lewis

**Affiliations:** St John’s Institute of Dermatology, Guy’s and St Thomas’ NHS Foundation Trust, London, UK; St John’s Institute of Dermatology, School of Basic and Medical Biosciences, King’s College London, London, UK; Dermatology Surgery and Laser Unit, St John’s Institute of Dermatology, Guy’s and St Thomas’ NHS Foundation Trust, London, UK; St John’s Institute of Dermatology, Guy’s and St Thomas’ NHS Foundation Trust, London, UK

## Abstract

Acquired vulval lymphangiectasia is a rare and often misdiagnosed condition with significant impacts on quality of life. We report a case of idiopathic vulval lymphangiectasia successfully treated with carbon dioxide (CO_2_) laser ablation, resulting in durable symptom resolution and restored quality of life. This highlights CO_2_ laser as a valuable, minimally invasive therapeutic option for this challenging disorder.

What is already known about this topic?

- Acquired vulval lymphangiectasia is a rare disorder caused by disruption of lymphatic drainage and can significantly impair quality of life.
- Management is challenging, with limited evidence to guide treatment and few data on long-term outcomes.

What does this study add?

- This case demonstrates sustained clinical improvement for over 10 years following CO₂ laser ablation in idiopathic acquired vulval lymphangiectasia.
- Carbon dioxide laser therapy may provide a durable, minimally invasive treatment option for selected patients with focal disease.

Acquired vulval lymphangiectasia (AVL), also termed acquired lymphangioma circumscriptum, is a rare and under-recognized disorder caused by disruption of local lymphatic drainage.^1^ It manifests as clusters of translucent or serosanguinous vesicles on the vulva, often misdiagnosed as viral warts, molluscum contagiosum or other dermatoses.^2^ It can result from previous pelvic surgery or radiotherapy, and can be associated with infection, Crohn disease, malignancy and trauma.^3^ In the absence of an identifiable trigger, they are termed idiopathic, but these cases are exceptionally rare. Patients typically experience swelling, discomfort, recurrent cellulitis and discharge from affected areas.^3^ This can have a significant impact on quality of life, particularly with regard to sexual function and avoidance of intimacy. Despite its impact, AVL remains under-reported, with no established guidelines for diagnosis or treatment. We present a case of idiopathic AVL successfully managed with carbon dioxide (CO_2_) laser ablation, highlighting both diagnostic complexity and therapeutic success.

## Case report

**Figure 1:**
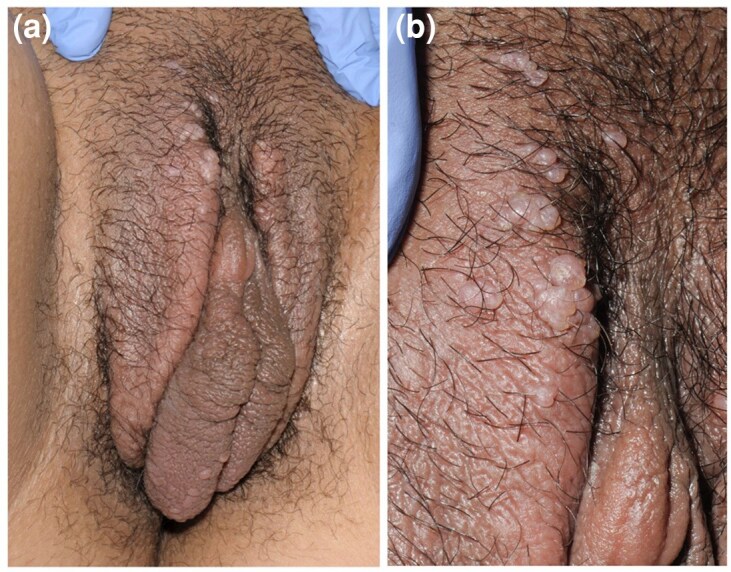
Clinical images of acquired vulval lymphangiectasia taken in 2013 before treatment showing (a) oedema to the labia majora and minora. (b) A magnified view showing the overlying papulovesicular lesions.

A 34-year-old woman with Fitzpatrick skin phototype IV and a normal body habitus presented with a 12-month history of painful vulval swelling. On examination, multiple papules were noted involving the labia majora and minora (Figure 1). She attributed the onset to the use of a depilatory cream in the pubic area but had no visible reaction on the skin after using this. She experienced monthly flares characterized by localized erythema, ­significant watery drainage, high-grade fever and debilitating discomfort. Histopathology of a skin biopsy revealed multiple dilated lymphatic vessels within the papillary dermis, confirming the diagnosis of lymphangiectasia. Epidermal acanthosis and mild perivascular inflammation were noted, with no evidence of malignancy or granulomatous disease. A comprehensive workup revealed negative antinuclear antibody and extractable nuclear antigen testing, as well as normal inflammatory markers (C-reactive protein and erythrocyte sedimentation rate) and faecal calprotectin (<50 µg g^–1^). An angiotensin-converting enzyme test and tuberculosis interferon-γ release assay were also negative. She had no history of herpes simplex infection and swabs for herpes simplex virus polymerase chain reaction during an acute flare were negative. Magnetic resonance imaging of the pelvis demonstrated mild hyperintensity on short tau inversion recovery sequences within the labia majora, consistent with dermal oedema, and a stable 3.8-cm right subserosal fibroid. This fibroid was relatively small and not considered to exert any significant local pressure effect on lymphatic drainage. No pelvic lymphadenopathy, deep lymphatic malformations or masses were seen. Abdominal ultrasound showed no intra-abdominal pathology. These investigations, together with the lack of surgical or radiation history, supported a diagnosis of acquired vulval lymphangiectasia (AVL).

**Figure 2:**
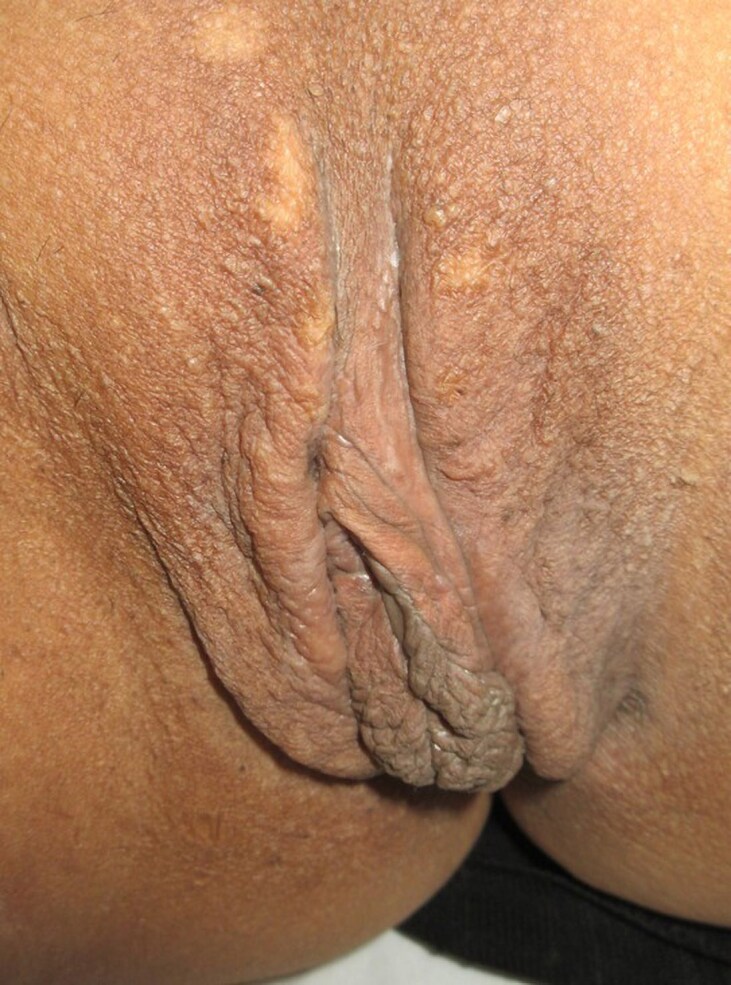
Post-treatment image demonstrating sustained clinical improvement following carbon dioxide laser therapy at long-term follow-up (2025).

The patient was commenced on long-term erythromycin (250 mg twice daily) to prevent the recurrent cellulitis, which provided partial relief. However, the lesions continued to affect her ability to engage in sexual activity. A Dermatology Life Quality Index assessment yielded a score of 13, indicating a very large impact on her quality of life. She also reported that hair removal, which was culturally important, appeared to exacerbate her symptoms; however, no clear causal relationship could be established. She noted that the swelling and episodes of leakage were much worse during pregnancy, likely due to the gravitational effects of the gravid uterus. Given the focal nature of the disease, she underwent staged carbon dioxide (CO_2_) laser ablation with the Lumenis UltraPulse® CO_2_ laser (Lumenis, High Wycombe, UK). Topical anaesthaesia was achieved using EMLA® cream (Aspen Pharma, Dublin, Ireland) applied for 45 min under occlusion prior to the procedure to minimize discomfort while allowing for careful control of ablation depth. Laser parameters were set at 175 mJ with a computer pattern generator setting of 1–2–5. Ablation was performed in sequential passes, with the surface wiped between passes, until the dermal layer was reached, as indicated by characteristic tissue response. This approach permitted controlled removal of affected tissue while minimizing collateral thermal injury and scarring. The patient underwent two staged CO_2_ laser sessions in 2013, resulting in near-complete resolution. She remained satisfied with the outcome and required no further treatment over 10 years, until 2026, when she re-presented for management of a single persistent lesion on the right labium majus. A further CO_2_ laser treatment to address this lesion is planned. At follow-up, she remained otherwise asymptomatic with no new lesions and reported sustained improvement in quality of life (Figure 2).

## Discussion

The management of AVL is challenging. Conservative strategies, including antibiotics, compression and drainage, offer temporary symptom relief but do not address the underlying lymphatic pathology.^1^ Historically, surgical has been the mainstay for extensive disease but is associated with high recurrence rates and considerable morbidity.^4^ CO_2_ laser ablation represents an effective and minimally invasive alternative. It vaporizes superficial dermal lymphatics, sealing dilated channels while preserving ­surrounding structures. Potential adverse effects include post­inflammatory hypopigmentation, particularly in patients with darker skin phototypes, which should be considered during patient counselling. Successful use of CO_2_ laser in a patient with AVL secondary to a large uterine fibroid, with resolution after hysterectomy and two laser sessions, has been described.^5^ In settings where CO_2_ laser therapy is unavailable or cost-prohibitive, alternative ablative modalities including electrocautery (hyfrecation), electrodessication and cryotherapy have also been reported.^3^ Sclerotherapy, including intralesional bleomycin, has been used in the management of lymphatic malformations more broadly; however, its use in vulval lymphangiectasia has not been well described.^6^

While CO_2_ laser therapy has been described previously in the management of vulval lymphangiectasia,^3,7^ long-term outcomes following CO_2_ laser ablation in idiopathic AVL have not been well characterized. Our patient had no identifiable predisposing factor, pelvic abnormality or systemic disease. Her disease course was complicated by recurrent infection, failed conservative management and significant quality-of-life impairment, highlighting the therapeutic and psychosocial challenges of this rare condition. Although the patient associated symptom exacerbation with hair removal, there is limited evidence in the literature supporting a causal role, and this remains speculative. CO_2_ laser is widely accessible and allows precise targeting of affected areas. Its efficacy, tolerability and favourable cosmetic outcomes make it a valuable addition to the therapeutic armamentarium. Given the potential for diagnostic delay, recurrent infection and significant impact on sexual function, coordinated multidisciplinary input may help optimize clinical and quality-of-life outcomes in selected patients.

## Data Availability

This article reports a single patient case; no datasets were generated or analysed. All relevant clinical information is contained within the manuscript.
